# Urinary Profiles of Exosomal LINE-1 mRNA and Associated miRNAs in Non-Small-Cell Lung Cancer

**DOI:** 10.3390/cells15131231

**Published:** 2026-07-07

**Authors:** Abeer A. I. Hassanin, Kenneth S. Ramos

**Affiliations:** 1 Center for Genomic and Precision Medicine, Texas A&M Institute of Biosciences and Technology, Texas Medical Center, Houston, TX 77030, USA; ahassanin@tamu.edu; 2 Department of Animal Wealth Development, Faculty of Veterinary Medicine, Suez Canal University, Ismailia 41522, Egypt

**Keywords:** urine exosomes, NSCLC, LINE-1, miRNAs, diagnostic performance

## Abstract

**Highlights:**

**What are the main findings?**

- Urine-derived exosomes from NSCLC patients exhibit elevated levels of LINE-1 ORF1/ORF2 mRNA and multiple cancer-associated miRNAs compared with healthy controls.
- The molecular cargo of urine-derived exosomes largely mirrors plasma exosomal profiles and correlates with clinicopathologic characteristics of NSCLC.

**What are the implications of the main findings?**

- Urine exosomal RNA profiling provides a complementary, noninvasive, and readily accessible approach for the evaluation of NSCLC patients.

**Abstract:**

Lung cancer remains the leading cause of cancer-related mortality worldwide in both males and females. Despite recent advances in precision-targeted therapeutics, mortality rates remain high, largely due to delayed diagnoses when curative interventions are no longer feasible. Recent studies from our group demonstrated that the LINE-1 mRNA and associated miRNA cargo of plasma exosomes can be used as sensitive and specific diagnostic and prognostic biomarkers of non-small-cell lung cancer (NSCLC). Because exosomes from various cancer types can be detected in urine, we extended our investigation to examine these analytes in urine exosomes from NSCLC patients. LINE-1 ORF1 and ORF2 mRNA levels, along with miR-21-5p, miR-126-3p, miR-210-3p, miR-221-3p, Let-7b-5p, miR-146a-5p, miR-222-3p, miR-9-5p, and miR-1277-5p, were higher in urine exosomes from NSCLC patients compared to healthy controls. The cargo of urine-derived exosomes often mirrored that of plasma exosomes and correlated with several clinicopathologic characteristics. The strong predictive performance of urine exosomal RNAs distinguishing NSCLC patients from controls suggests these measurements may serve as a complementary and readily accessible source for noninvasive assessment of patients with NSCLC.

## 1. Introduction

Lung cancer ranks as the second most prevalent cancer and the leading cause of cancer-related mortality in both males and females worldwide [1]. Non-small-cell lung cancer (NSCLC) accounts for approximately 85% of all lung cancer cases [2]; and histologically classified into four subtypes, with lung adenocarcinoma (LUAD) and squamous cell lung carcinoma (SQCLC) are the most common. Recent studies suggest that SQCLC and LUAD should be considered biologically distinct tumors and managed with subtype-specific therapeutic strategies [3]. However, current diagnostic approaches do not always reliably distinguish between these two subtypes.

Recent work from our group demonstrated that Long Interspersed Element-1 (LINE-1) DNA and mRNA are present in circulating plasma-derived exosomes [4,5]. Exosomes are extracellular vesicles ranging in diameter from 30 to 150 nm and released into biological fluids by nearly all cell types. Their cargo includes a diverse array of functional biomolecules, such as proteins, nucleic acids, and lipids, many of which are being proposed as biomarkers for the diagnosis and monitoring of various diseases, including lung cancer [6,7,8]. Notably, lung-derived exosomes constitute a substantial proportion of the circulating exosomal population in the bloodstream [9,10]. While most studies to date have focused on plasma-derived exosomes, urinary exosomes have also attracted increasing attention. The noninvasive and convenient nature of urine collection makes urine an attractive biofluid for studying systemic diseases [11,12]. Importantly, circulating exosomes originating from lung tumors have been detected in urine [13]. The ability of exosomes to cross physiological barriers, likely due to their lipid-based membrane structure and nanoscale size, may contribute to their widespread distribution across multiple body fluids.

The expression of LINE-1 retroelements has been strongly associated with cancer initiation and progression [14], with the presence of enzymatically active LINE-1 components within extracellular vesicles well documented [4,15,16]. The potential clinical utility of LINE-1 analytes in plasma-derived exosomes from NSCLC patients has been demonstrated in studies showing robust discrimination between both LUAD and SQCLC patients and healthy controls [17]. In addition, LINE-1-associated microRNAs (miRNAs) have been characterized and shown to modulate oncogenic signaling in naïve recipient cells [18]. Tumor-derived exosomes frequently recapitulate the profiles of their cells of origin, thereby providing a reliable source of biomarkers for personalized cancer diagnosis and therapeutic monitoring [4,19].

In the present study, we examine LINE-1 analytes and associated miRNAs in urine-derived exosomes from NSCLC patients. Our findings demonstrate that urinary levels of these biomarkers mirror those previously observed in plasma-derived exosomes, suggesting that urine may serve as a complementary and noninvasive biofluid for the longitudinal monitoring of NSCLC.

## 2. Material and Methods

### 2.1. Urine Samples and Patient Records

Thirty-six human urine samples, each 1 mL, were obtained from BioIVT (Westbury, NY, USA) in K2EDTA or NaCit vacutainer tubes. Twelve samples were from ostensibly healthy individuals (Cont.), twelve from patients diagnosed with SQCLC, and twelve from patients diagnosed with LUAD.

### 2.2. Exosome Isolation and Characterization

Exosomes were extracted from urine samples using the exoEasy Maxi Kit (Qiagen, catalog number: 76064, Germantown, MD, USA), adhering to the manufacturer’s protocol after centrifugation for 10 min at 4 °C and 16,000× *g* to eliminate residual cells, cell debris, apoptotic bodies, and nuclei. Physical characterization of isolated exosomes was conducted using the NanoSight NS300 instrument (NTA) (Malvern Panalytical Inc., Columbia, MD, USA). Western blot analysis was conducted for evaluation of conventional exosomal markers Alix, CD-9, and the negative marker of exosomes, calnexin.

### 2.3. Real-Time PCR Analyses for Exosomal LINE mRNAs and Associated miRNAs

Exosomal RNA was extracted using the TRIzol reagent (Invitrogen, Carlsbad, CA, USA). The concentration and quality of RNAs were measured using Gen 5^TM^ data analysis software (BioTek Instruments, Inc., Winooski, VT, USA). The Reverse Transcription System (Promega Corporation, Madison, WI, USA) was used to synthesize cDNA from equal RNA inputs across all samples to minimize technical variability (100 ng of each RNA sample), per the manufacturer’s protocol. The CFX96 Touch Real-Time PCR Detection System (Bio-Rad Laboratories, Inc., Hercules, CA, USA) was used to assess exosomal LINE-1 ORF1 and ORF2 mRNA or miRNAs using Power SYBR^®^ Green Master Mix (Applied Biosystems, Thermo Fisher Scientific, Waltham, MA, USA). All reactions included an initial denaturation at 95 °C for 10 min 40 cycles of denaturation at 95 °C for 15 s followed by annealing at 60 °C for 1 min. Each experiment was conducted three times, and each sample was run in triplicate each time. 18s rRNA was used to normalize both LINE-1 ORF1 and ORF2 mRNA levels, while U6 was used as a reference gene to normalize exosomal miRNA levels.

### 2.4. Diagnostic Utility of Urine Exosomal LINE-1 mRNA Level Profiles

Receiver operating characteristic (ROC) curve analysis was employed to assess the diagnostic and prognostic performances of urine exosomal LINE-1 analytes in NSCLC. The most relevant cut-off values were established using the highest Youden index. *p* values of 0.05 or lower were considered significant, and 95% confidence intervals (CIs) were calculated.

### 2.5. Statistical Analyses

GraphPad Prism, version 9.5.0 (GraphPad Software, San Diego, CA, USA), was used for the analyses. To measure differences in urine exosomal miRNA levels between ostensibly healthy controls and patients with SQCLC or LUAD, ANOVA and Tukey’s HSD test were used. To compare the levels of LINE-1 and miRNAs amongst subgroups, unpaired *t*-test analysis was employed. Grubbs’s test was used to identify outliers.

## 3. Results

### 3.1. Cohort Demographics

The clinical and demographic characteristics of the study participants are summarized in Table 1. Equal numbers of female and male subjects with comparable mean ages were included in each study group. The proportion of non-smokers was higher in the LUAD group compared with the SQCLC group, while the numbers of former and current smokers were similar between the two NSCLC subtypes. Most subjects diagnosed with SQCLC presented at later stages of disease compared with LUAD patients; however, demographic information was unavailable for many participants. When tumor size data were available, comparable tumor size distributions were observed between the two cancer groups. Lymph node involvement was also similar between SQCLC and LUAD patients. Most participants did not exhibit disseminated metastatic disease at the time of sample collection.

### 3.2. Exosome Characterization

**Figure 1:**
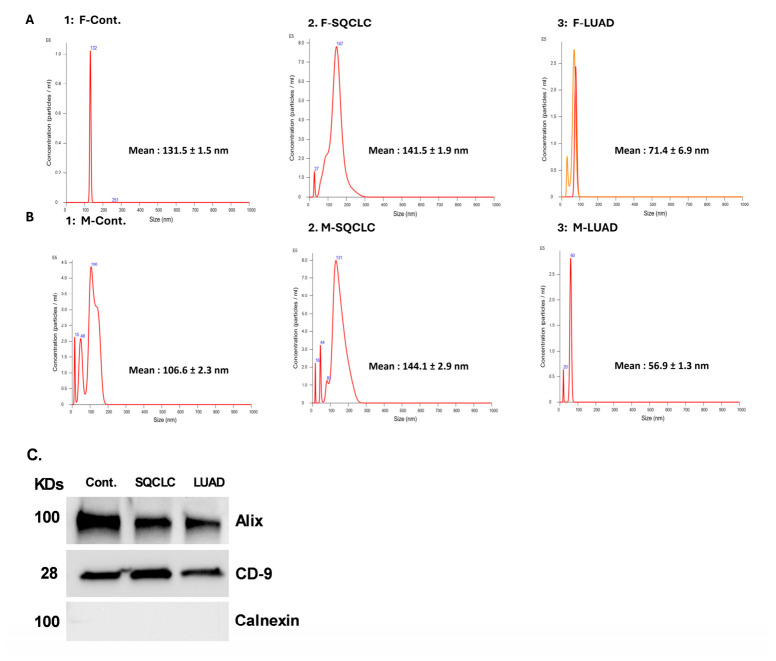
Characterization of urine-derived exosomes from controls and NSCLC patients. (**A**,**B**) Representative nanoparticle tracking analysis (NTA) profiles showing exosome size distribution. (**C**) Western blot detection of the exosomal markers Alix and CD9 and absence of the endoplasmic reticulum marker calnexin. Molecular weight markers are indicated in kilodaltons (kDa).

Urine-derived exosomes from ostensibly healthy individuals and patients with SQCLC and LUAD were characterized using nanoparticle tracking analysis (NTA). The analysis confirmed that exosomes from all groups were within the expected size range of extracellular vesicles (30–150 nm) (Figure 1A,B). Exosomes from LUAD patients were slightly smaller than those from SQCLC patients or healthy controls, but these differences were not statistically significant. Western blot analysis confirmed the identity of the isolated vesicles, with the canonical exosomal markers Alix and CD9 detected in all samples, while calnexin, an endoplasmic reticulum-associated protein often used as a negative control, was not detected (Figure 1C).

### 3.3. Exosomal LINE-1 ORF1/ORF2 mRNA Levels Correlate with Tumor Clinical Characteristics

**Figure 2:**
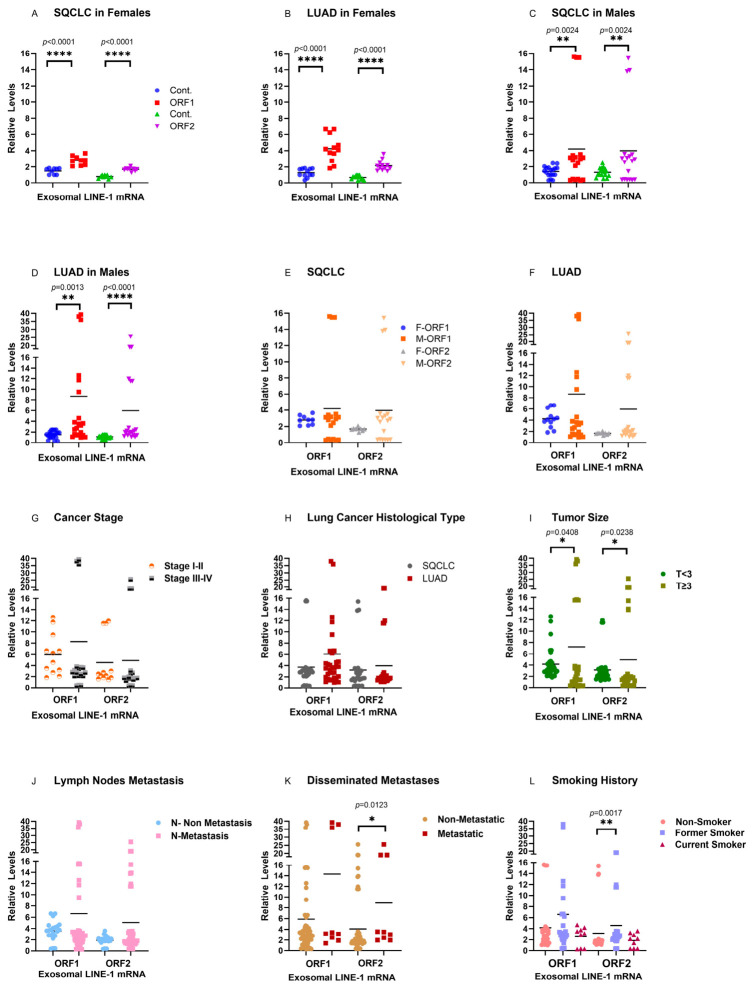
Exosomal LINE-1 ORF1 and ORF2 mRNA expression in NSCLC. (**A**,**B**) Female patients with SQCLC or LUAD showed significantly elevated ORF1 and ORF2 mRNA levels compared with controls. (**C**,**D**) Male patients with SQCLC or LUAD also exhibit significantly increased LINE-1 mRNA levels relative to controls. (**E**,**F**) No significant differences were observed between male and female patients. (**G**–**L**) Correlations between LINE-1 mRNA levels and clinicopathologic characteristics including cancer stage, tumor size, lymph node metastasis, disseminated metastasis, histological subtype, and smoking status. Tumors larger than or equal to three showed significantly higher levels of LINE-1 ORF1 and ORF2 than smaller-sized tumors. Metastatic NSCLC tumors had significantly higher levels of LINE-1 ORF2 mRNA than non-metastatic tumors. Statistical significance was indicated as follows: *p* < 0.05 (*), *p* < 0.01 (**), and *p* < 0.0001 (****).

Elevated levels of LINE-1 ORF1 and ORF2 mRNA were detected in both female and male patients with SQCLC and LUAD compared with healthy controls (Figure 2A–D). Comparable levels of ORF1 and ORF2 transcripts were observed between female SQCLC and LUAD samples (Figure 2A,B). A similar pattern was observed in male patients (Figure 2C,D), although greater variability was noted among several tumor samples. A comparison between sexes revealed no significant differences in ORF1 or ORF2 mRNA levels for either histological subtype (Figure 2E,F). Elevated LINE-1 analyte levels were observed across all clinicopathologic parameters evaluated (Figure 2G–L). However, statistically significant differences between tumor and control groups were observed only for tumor size (Figure 2I), disseminated metastasis (Figure 2K), and smoking status (Figure 2L), where significantly higher ORF2 levels were observed in former smokers compared with non-smokers. No sex-specific differences were observed when the data were stratified by the six clinicopathologic parameters analyzed. To evaluate the potential impact of urine dilution on exosomal LINE-1 levels, secondary analyses were conducted to normalize samples by exosome particle counts rather than RNA concentration. Profiles of LINE-1 expression were remarkably similar and consistent between the two approaches for both females and males across the two major NSCLC subtypes. Modest differences in sample dispersion were observed, suggesting that urine dilution may influence the overall distribution of exosomal LINE-1 levels (Supplementary Figure S1).

### 3.4. Diagnostic and Prognostic Utility of Exosomal LINE-1 ORF1 and ORF2 mRNA Levels

**Figure 3:**
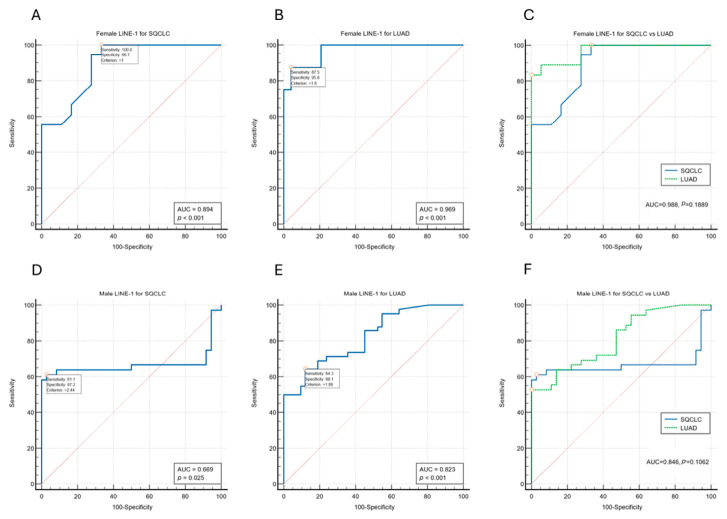
ROC curve analysis of exosomal LINE-1 mRNAs in patients with NSCLC. The diagnostic efficacy of (**A**) females with SQCLC versus controls; (**B**) females with LUAD versus controls; (**C**) females with SQCLC compared to LUAD; (**D**) males with SQCLC versus controls; (**E**) males with LUAD versus controls; and (**F**) males with SQCLC versus those with LUAD. The red line represents the line of no-discrimination, The circle symbol indicates the optimal cutoff value selected based on the highest diagnostic accuracy (Youden index).

Receiver operating characteristic (ROC) curve analysis demonstrated strong diagnostic performance of urinary exosomal LINE-1 mRNA levels. Among female subjects, combined ORF1/ORF2 measurements distinguished SQCLC patients from healthy controls (AUC = 0.894, sensitivity 100%, specificity 66.7%) (Figure 3A). Even stronger diagnostic performance was observed for distinguishing LUAD patients from controls (AUC = 0.969, sensitivity 87.5%, specificity 95.8%) (Figure 3B). These analytes also demonstrated strong discrimination between the two NSCLC histological subtypes (AUC = 0.988) (Figure 3C). Among male subjects, exosomal LINE-1 mRNA distinguished SQCLC patients from controls (AUC = 0.669, sensitivity 61.1%, specificity 97.2%) (Figure 3D). Diagnostic performance for LUAD was higher (AUC = 0.823, sensitivity 64.3%, specificity 88.1%) (Figure 3E). These markers also differentiated SQCLC from LUAD (AUC = 0.846) (Figure 3F).

**Figure 4:**
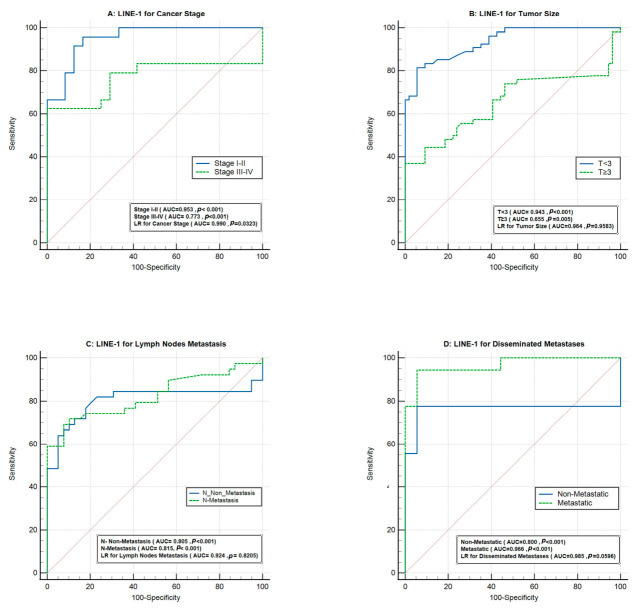
Prognostic utility of urinary exosomal LINE-1 mRNAs distinguishing (**A**) cancer stage, (**B**) tumor size, (**C**) lymph node metastasis, and (**D**) disseminated metastases. The red line represents the line of no-discrimination.

Stratified analyses further demonstrated the prognostic value of urinary exosomal LINE-1 mRNA levels (Figure 4). These markers effectively distinguished early-stage (I–II) and late-stage (III–IV) NSCLC patients from controls (Figure 4A), with AUC values of 0.963 and 0.773, respectively (*p* < 0.001). Similarly, LINE-1 mRNA accurately distinguished small tumors (T < 3) from large tumors (T ≥ 3) (*p* < 0.001). Logistic regression analysis demonstrated excellent discrimination between these groups (AUC = 0.964) (Figure 4B). Evaluation of lymph node metastasis also showed strong discriminatory performance (AUC = 0.924), while analysis of disseminated metastasis demonstrated excellent predictive accuracy (AUC = 0.905) (Figure 4C,D). Collectively, these findings demonstrate the potential of urinary exosomal LINE-1 mRNA as a sensitive diagnostic and prognostic biomarker for NSCLC (Table 2).

### 3.5. miRNA Profiles in NSCLC Urine Exosomes

**Figure 5:**
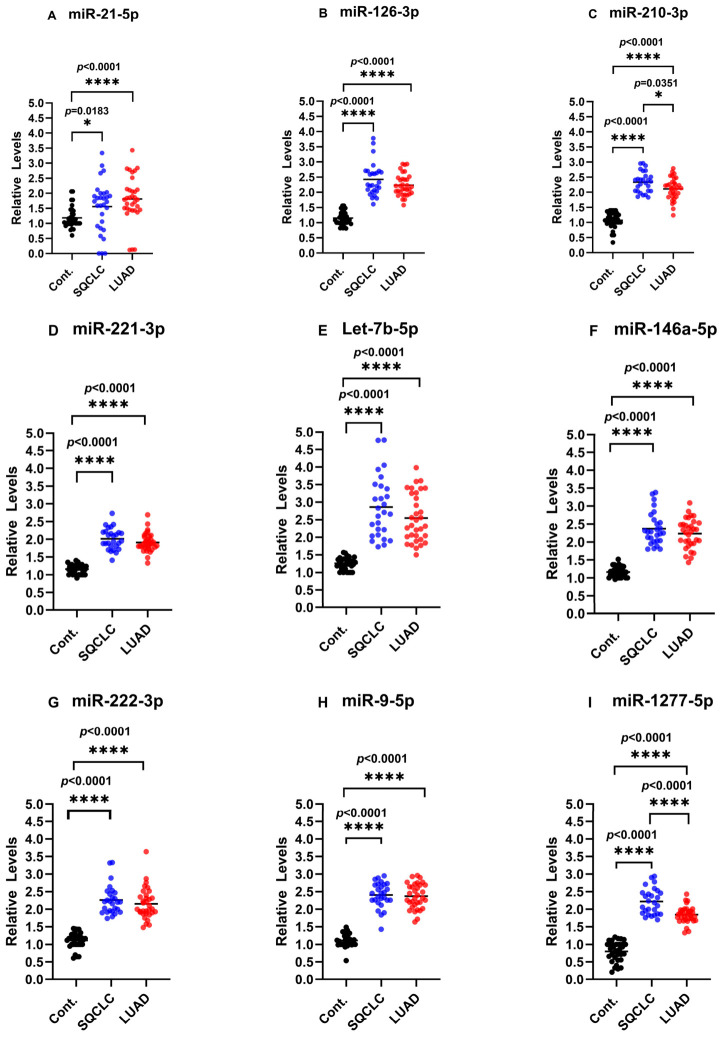
Relative fold change in the nine exosomal miRNAs in healthy control group (cont.) compared to SQCLC and LUAD; miR-21-5p (**A**), miR-126-3p (**B**), miR-210-3p (**C**), miR-221-3p (**D**), Let-7b-5p (**E**), miR-146a-5p (**F**), miR-222-3p (**G**), miR-9-5p (**H**), and miR-1277-5p (**I**). Statistical significance was indicated as follows: *p* < 0.05 (*), and *p* < 0.0001 (****).

Nine miRNAs, namely, miR-21-5p, miR-126-3p, miR-210-3p, miR-221-3p, Let-7b-5p, miR-146a-5p, miR-222-3p, miR-9-5p, and miR-1277-5p, were detected in all groups (Figure 5A–I). All miRNAs exhibited significantly elevated levels in both NSCLC subtypes compared to the controls. Sex-specific analyses (Supplementary Figures S2 and S3) revealed significant differences in both female and male patients with SQCLC and LUAD relative to the controls. Among females, miR-210-3p and let-7b-5p were significantly higher in SQCLC compared with LUAD (*p* = 0.0227 and *p* = 0.0043, respectively). Among males, miR-221-3p and miR-1277-5p were significantly elevated in SQCLC relative to LUAD (*p* = 0.0453 and *p* < 0.0001).

### 3.6. Exosomal miRNAs and Clinicopathologic Characteristics

**Figure 6:**
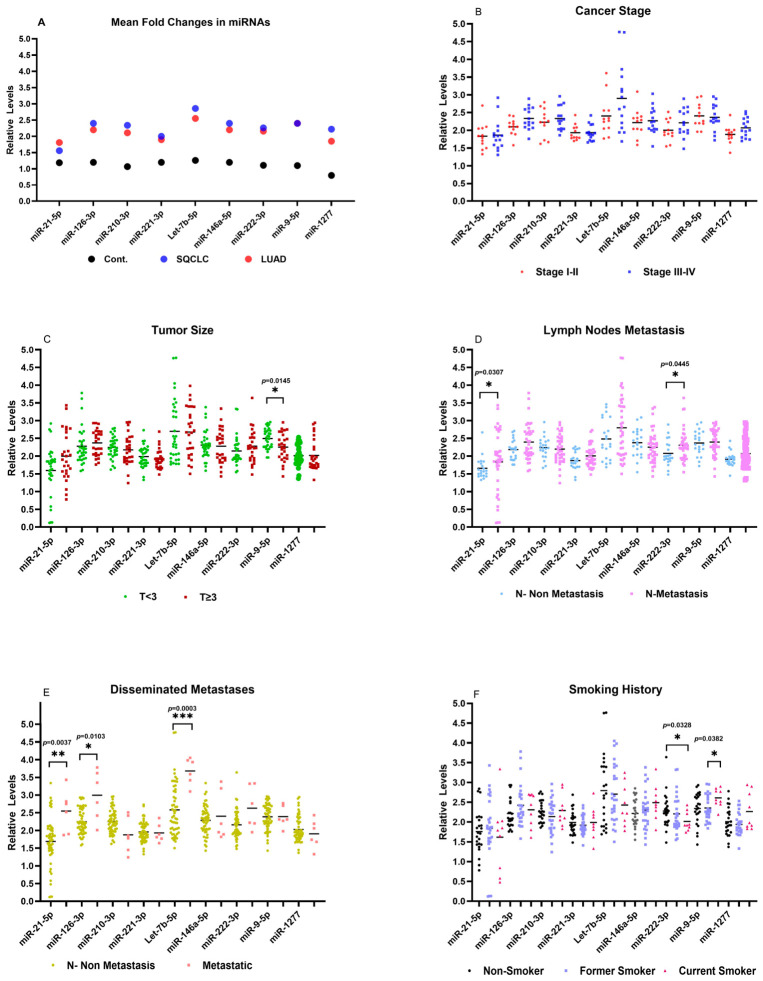
(**A**) Mean fold exosomal miRNA changes in ostensibly healthy controls and patients with SQCLC and LUAD. (**B**) Levels of the nine microRNAs did not differ significantly with respect to cancer stage. (**C**) NSCLC patients with T < 3 showed significantly elevated levels of exosomal miR-9-5p (*p* = 0.0145) compared to larger-sized tumors. (**D**) NSCLC patients with lymph node metastasis showed significantly higher levels of miR-21-5p (*p* = 0.0307) and miR-222-3p (*p* = 0.0445) than those without metastasis. (**E**) Disseminated metastatic NSCLC tumors exhibited significantly higher levels of exosomal miR-21-5p (*p* = 0.0037), miR-126-3p (*p* = 0.0103), and Let-7b-5p (*p* = 0.0003) than non-metastatic subjects. (**F**) Non-smoker NSCLC patients showed significantly higher levels of miR-222-3p (*p* = 0.0326) compared to current smokers. However, current smoker patients showed significantly higher levels of miR-9-5p (*p* = 0.0382) compared to former smokers. Statistical significance was indicated as follows: *p* < 0.05 (*), *p* < 0.01 (**), and *p* < 0.001 (***).

Correlation analyses between exosomal miRNAs and clinicopathologic characteristics are shown in Figure 6. No significant differences were observed between miRNA levels and cancer stage (Figure 6B). However, miR-9-5p levels were significantly higher in smaller tumors (Figure 6C). Additionally, miR-21-5p and miR-222-3p were significantly elevated in tumors with lymph node metastasis (Figure 6D). In cases of disseminated metastasis, miR-21-5p, miR-126-3p, and let-7b-5p were significantly increased (Figure 6E). Smoking status also influenced miRNA expression patterns. miR-222-3p levels were significantly higher in non-smokers compared with current smokers, whereas miR-9-5p levels were elevated in current smokers relative to former smokers (Figure 6F).

## 4. Discussion

Recent studies from our group have demonstrated the strong performance of plasma exosomal LINE-1 analytes and associated miRNAs as diagnostic and prognostic biomarkers for NSCLC [17,20]. Here, we extend these findings by evaluating urine-derived exosomes as a complementary, noninvasive biomatrix for the assessment of lung cancer patients. This study was motivated by earlier reports showing that circulating lung cancer-derived exosomes can be detected in urine [13]. However, the precise mechanisms by which lung cancer-derived exosomes enter the urinary space remain unclear [13].

Urine-derived exosomes exhibited patterns that closely mirrored those observed in plasma exosomes, with elevated levels of LINE-1 ORF1 and ORF2 mRNA, as well as miR-21-5p, miR-126-3p, miR-210-3p, miR-221-3p, Let-7b-5p, miR-146a-5p, miR-222-3p, miR-9-5p, and miR-1277-5p in NSCLC patients compared to healthy controls. However, the levels of LINE-1 analytes and associated miRNAs in urine exosomes were consistently lower than those observed in plasma exosomes, likely reflecting dynamic changes in exosomal cargo during vesicular trafficking throughout the body. Interestingly, urine exosomal LINE-1 ORF2 mRNA levels were higher in former smokers compared with non-smokers, while no significant differences were noted between current smokers and non-smokers. Given that cigarette smoking induces long-lasting epigenetic alterations that persist after smoking cessation, including changes in DNA methylation and LINE-1 regulation [21,22], the ORF2 findings may reflect cumulative and persistent smoking-related molecular effects rather than current smoking status alone.

While plasma exosomes demonstrated higher overall diagnostic sensitivity and specificity, particularly for SQCLC [17], urinary exosomes showed modestly improved performance distinguishing LUAD patients from controls, possibly reflecting shifting dynamics in exosomal cargo between the two biofluids.

LINE-1 mRNA encodes two well-characterized proteins: open reading frame 1 protein (ORF1p) and open reading frame 2 protein (ORF2p). ORF1p is a 40 kDa polypeptide that forms a homotrimeric structure and functions as a nucleic acid chaperone [23,24]. ORF2p is a 150 kDa multidomain protein with endonuclease [25] and reverse transcriptase activities [26]. Together, ORF1p and ORF2p facilitate retrotransposon mobilization to new genomic sites and are associated with substantial alterations in chromatin organization and genomic instability. Studies by Ardeljan et al. [27] and Sciamanna et al. [28] further demonstrated that LINE-1-encoded reverse transcriptase activity may be linked to cancer initiation, supporting the use of LINE-1 methylation status, RNA transcripts, and protein expression as biomarkers for cancer detection. Consistent with these findings, blood-based studies have shown that individuals with suspicious lung nodules detected by CT imaging exhibit higher serum levels of ORF1p compared to those with negative scans [29].

The miRNA cargo of exosomes complements the oncogenic activity of LINE-1 by regulating key signaling pathways involved in tumor progression, intercellular communication, and the tumor microenvironment [18,30]. In urinary exosomes, elevated levels of miR-21-5p and miR-222-3p were associated with lymph node metastasis, while disseminated metastatic tumors exhibited increased levels of miR-21-5p, miR-126-3p, and Let-7b-5p. Both miR-21-5p and miR-222-3p are well-established biomarkers of lymph node metastasis [31,32,33,34]. Increased miR-21 expression correlates with advanced clinical stage and metastasis, partly through the inhibition of PTEN, leading to enhanced tumor proliferation and invasion [35]. Additionally, overexpression of miR-21-5p and miR-222-3p has been linked to oxidative stress and mitochondrial apoptosis in lung cancer [36]. miR-21 also promotes tumor metastasis through both immune modulation and intrinsic cellular mechanisms. It suppresses anti-tumor immune responses, creates a permissive microenvironment for metastatic dissemination, and directly enhances the invasive potential of cancer cells [37]. Similarly, miR-222-3p facilitates metastasis by suppressing the tumor suppressor TRPS1, which normally inhibits the epithelial–mesenchymal transition (EMT) regulator ZEB1. This suppression leads to ZEB1 activation, induction of EMT, and increased tumor cell invasion and migration [30]. Although some studies have reported undetectable levels of exosomal miR-126 in NSCLC [38,39,40], others have observed elevated levels in both early- and late-stage disease [41]. The Let-7 miRNA family is classically described as tumor-suppressive. However, Let-7 family members may exhibit pro-tumorigenic activities, including promotion of cancer cell migration, invasion, chemoresistance, and expression of progression-associated genes depending on tumor microenvironment and disease context [42]. This may account for the paradoxical increase seen in urinary exosomes of NSCLC patients. In our study, miR-9-5p levels were significantly higher in smaller tumors, consistent with prior findings showing elevated miR-9 expression in lung cancer tissues compared to normal tissue [43], where it promotes tumor growth, invasion, and adhesion [44,45]. Additionally, miR-9 has been implicated in regulating the balance between inflammation and lymphangiogenesis [46]. Several studies have reported that miR-9-5p can exhibit context-dependent and stage-specific expression patterns in cancer [47,48]. In early tumor development, miR-9-5p has been associated with tumor growth, proliferation, and epithelial–mesenchymal transition (EMT) [48], while in more advanced stages, its expression may be reduced due to alterations in regulatory networks or clonal selection during tumor progression [49]. Unlike plasma exosomes, urinary miR-222-3p and miR-9-5p showed significant associations with smoking status. Another distinguishing feature of urinary exosomes was the enrichment of miR-1277-5p. This observation aligns with previous reports showing upregulation of miR-1277-5p in plasma-derived small extracellular vesicles from NSCLC patients, implicating roles in protein maturation and metabolic regulation [31]. Putative target genes and co-expression networks for the miRNAs identified in urine exosomes were previously described by our group [20]. Furthermore, we have shown that eight exosomal miRNAs identified in plasma from NSCLC patients modulate LINE-1-regulated oncogenic signaling in non-transformed human bronchial epithelial cells [18].

To our knowledge, this is the first report describing the potential application of urinary exosomal LINE-1 and associated miRNA cargo in the evaluation of NSCLC patients. Although this study is limited by a small sample size and the lack of an independent validation cohort, the findings provide preliminary evidence supporting the feasibility of urinary LINE-1 analytes for the diagnostic and prognostic evaluation of NSCLC. Importantly, the consistency of findings between plasma and urine exosomal measurements highlights the potential complementary roles of these two matrices in disease assessment. Nevertheless, our findings should be interpreted with caution as there is a need for validation in larger, independent cohorts. Future studies should quantitatively compare the dynamics of LINE-1 analytes and associated miRNAs in plasma and urine to further elucidate their biological relevance and potential clinical utility.

## Figures and Tables

**Table 1:** Demographic profiles of control and test groups.

Clinical Profile		Control (N = 12)	NSCLC	
			SQCLC (N = 12)	LUAD (N = 12)
Sex	Female	6	6	6
	Male	6	6	6
Age (Mean ± SE)		68.73 ± 1.21	68.73 ± 1.37	69 ± 1.65
Smoking Status	Never Smoker	N/A	3	6
	Former Smoker	N/A	4	4
	Current Smoker	N/A	2	1
	N/A	-	3	1
TNM Stage	I–II	-	0	4
	III–IV	-	4	1
	N/A	-	8	7
Tumor Size (T)	T < 3	-	5	7
	T ≥ 3	-	4	4
	N/A	-	3	1
Lymph Node Involvement	NO	-	2	6
	Yes	-	7	5
	N/A	-	3	1
Disseminated Metastasis	Non-Metastatic	-	8	10
	Metastatic	-	1	1
	N/A	-	3	1

N/A: not available; “-”: not applicable.

**Table 2:** Prognostic utility of urinary exosomal LINE-1 mRNA.

TNM Staging		Urine Exosomal LINE-1 mRNA			
		AUC (95% CI)	SENS	SPEC	Criterion
Cancer Stage	I–II	0.953 (0.850 to 0.993)	95.8	83.3	>1.56
	III–IV	0.773 (0.657 to 0.865)	0.60	97.1	>2.03
	I–II vs. III–IV	0.990 (0.907 to 1.0000	-	-	-
Tumor Size (T)	T < 3	0.943 (0.889 to 0.976)	80.3	95.5	>2.03
	T ≥ 3	0.655 (0.557 to 0.744)	37	100	>2.44
	T < 3 vs. T ≥ 3	0.964 (0.910 to 0.990)	-	-	-
Lymph Node Involvement (N)	NO	0.805 (0.700 to 0.886)	82.1	76.9	>1.7
	Yes	0.815 (0.745 to 0.873)	61.5	92.3	>1.88
	No vs. Yes	0.924 (0.842 to 0.972)	-	-	-
Disseminated Metastasis (M)	NO	0.800 (0.741 to 0.851)	60.2	95.4	>2.03
	YES	0.966 (0.845 to 0.998)	94.4	94.4	>1.88
	NO vs. Yes	0.985 (0.875 to 1.000)	-	-	-

“-” symbol indicates that the value is not applicable.

## Data Availability

The data presented in this study are available on request from the corresponding author.

## Supplementary Materials

The following supporting information can be downloaded at https://www.mdpi.com/article/10.3390/cells15131231/s1. Figure S1: Exosomal LINE-1 ORF1 and ORF2 mRNA expression in NSCLC. Figure S2: Relative fold change in expression levels of nine urine-derived exosomal miRNAs among female ostensibly healthy controls (cont.), squamous cell lung cancer (SQCLC) patients, and lung adenocarcinoma (LUAD) patients. Figure S3: Relative fold change in expression levels of nine urine-derived exosomal miRNAs among male ostensibly healthy controls (cont.), squamous cell lung cancer (SQCLC) patients, and lung adenocarcinoma (LUAD) patients.
