# Supplementary material for: Urinary Profiles of Exosomal LINE-1 mRNA and Associated miRNAs in Non-Small-Cell Lung Cancer
Source: Cells. 2026 Jul 7;15(13):1231. doi: 10.3390/cells15131231 (PMC13360329; doi:10.3390/cells15131231)
Supplement: Supplementary file 1 [file cells-15-01231-s001.zip › cells-4326030-supplementary.pdf]

Figures Legends:

Supplementary Figure S1: Exosomal LINE-1 ORF1 and ORF2 mRNA expression in NSCLC. (A–B) Female patients with SQCLC or LUAD, respectively; (C–D) Male patients with SQCLC or LUAD, respectively.

Supplementary Figure S2: Relative fold change in expression levels of nine urine-derived exosomal miRNAs among female ostensibly healthy controls (cont.) , squamous cell lung cancer (SQCLC) patients, and lung adenocarcinoma (LUAD) patients; A)miR-21-5p, B)miR-126-3p, C)miR-210-3p, D)miR-221-3p, E) Let-7b-5p, F) miR-146a-5p, G) miR-222-3p, H) miR-9-5p, I) miR-1277-5p. “1.0” denotes the calibrator control sample used as baseline for relative quantification and selected based on high RNA quality and stable endogenous control Ct values. All fold-change values were calculated using the ΔΔCt method relative to this sample.

Supplementary Figure S3: Relative fold change in expression levels of nine urine-derived exosomal miRNAs among male ostensibly healthy controls (cont.) , squamous cell lung cancer (SQCLC) patients , and lung adenocarcinoma (LUAD) patients; A)miR-21-5p, B)miR-126-3p, C)miR-210-3p, D)miR-221-3p, E) Let-7b-5p, F) miR-146a-5p, G) miR-222-3p, H) miR-9-5p, I) miR-1277-5p. “1.0” denotes the calibrator control sample used as baseline for relative quantification and selected based on high RNA quality and stable endogenous control Ct values. All fold-change values were calculated using the ΔΔCt method relative to this sample.

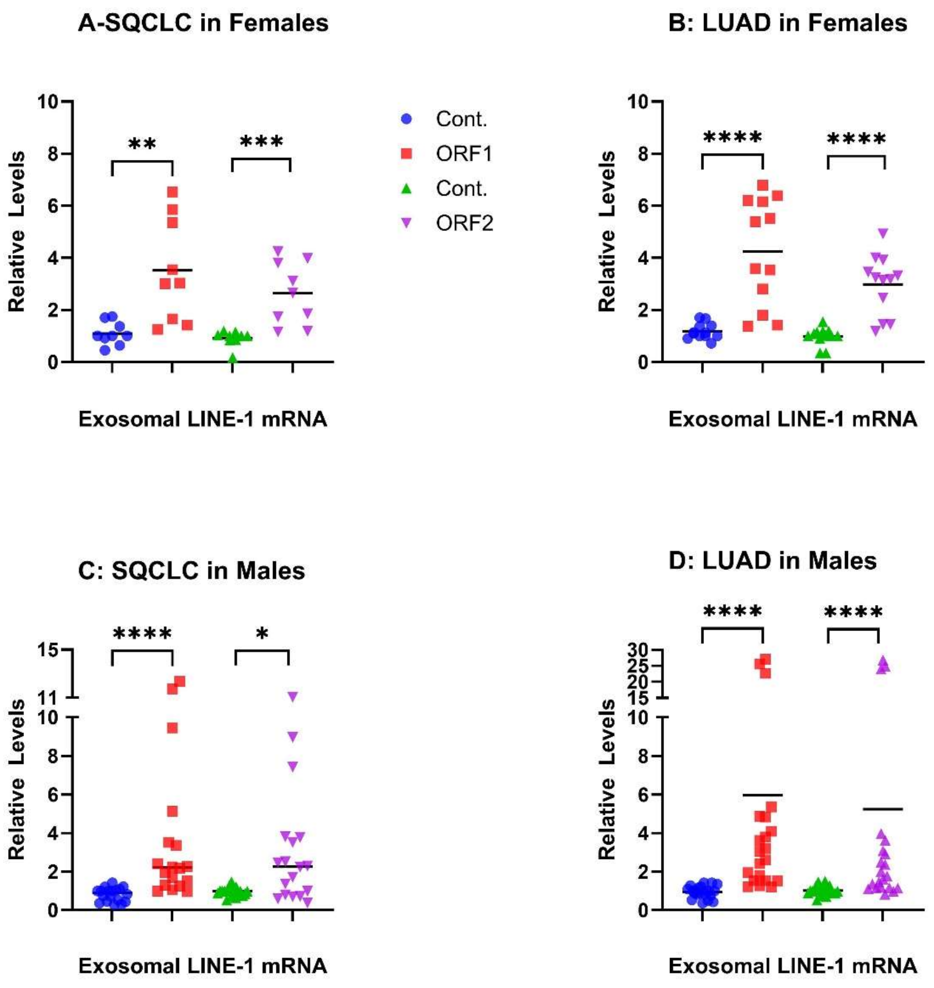

Supplementary Figure S1.

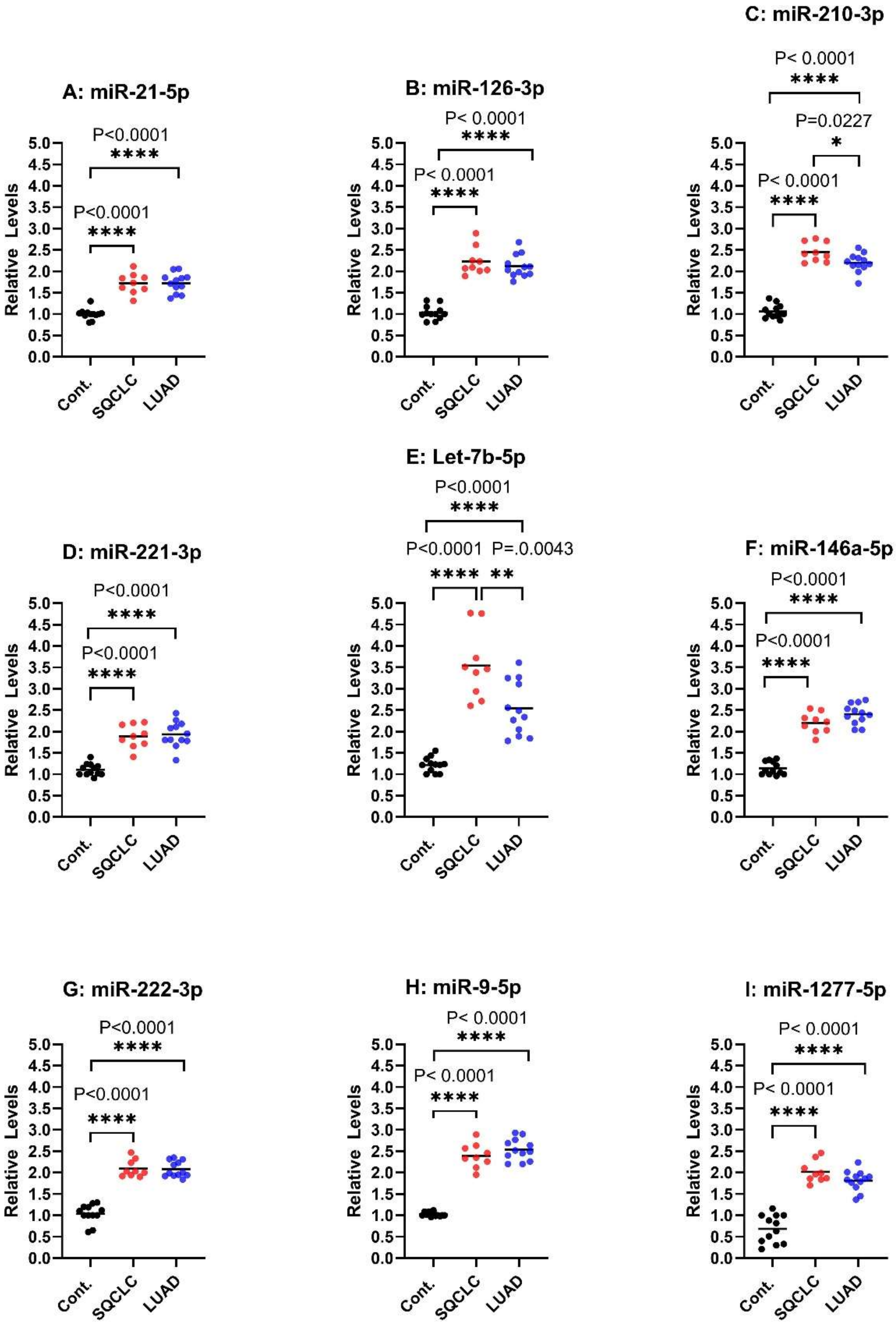

Supplementary Figure S2.

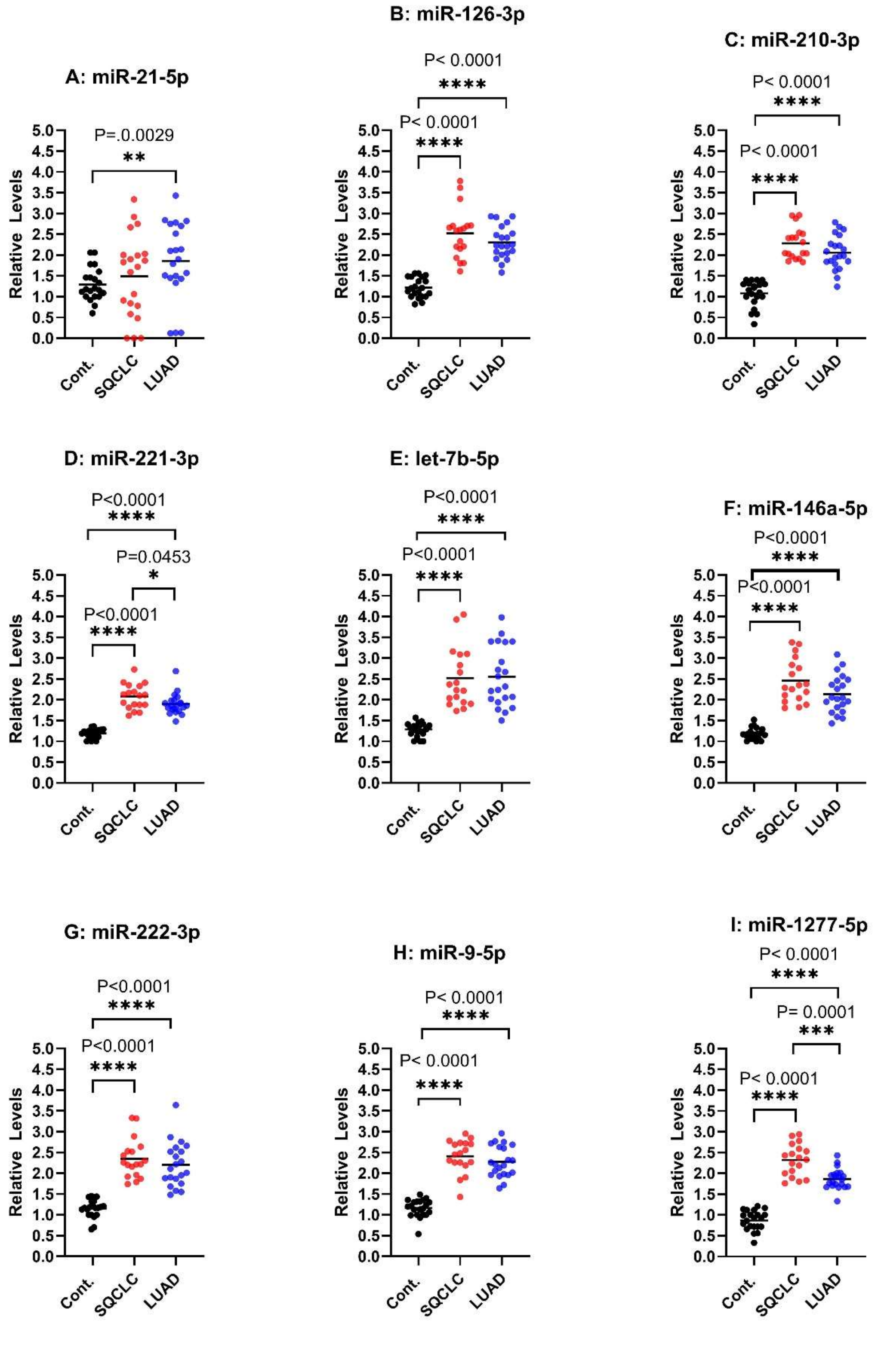

Supplementary Figure S3.
